# Kenya Psychosis-Risk Outcomes Study (KePROS): Development of an Accelerated Medicine Partnership Schizophrenia-Aligned Project in Africa

**DOI:** 10.1093/schizbullopen/sgae009

**Published:** 2024-05-04

**Authors:** Daniel Mamah, Victoria Mutiso, Christine Musyimi, Michael P Harms, Andrey P Anokhin, ShingShiun Chen, John Torous, Levi Muyela, Jerome Nashed, Yazen Al-Hosni, Arthur Odera, Alaina Yarber, Semyon Golosheykin, Masoomeh Faghankhani, Megan Sneed, David M Ndetei

**Affiliations:** Department of Psychiatry, Washington University Medical School, St. Louis, MO, USA; African Mental Health Research and Training Foundation, Nairobi, Kenya; African Mental Health Research and Training Foundation, Nairobi, Kenya; Department of Psychiatry, Washington University Medical School, St. Louis, MO, USA; Department of Psychiatry, Washington University Medical School, St. Louis, MO, USA; Department of Psychiatry, Washington University Medical School, St. Louis, MO, USA; Department of Psychiatry, Beth Israel Deaconess Medical Center at Harvard Medical School, Boston, MA, USA; African Mental Health Research and Training Foundation, Nairobi, Kenya; Department of Psychiatry, Washington University Medical School, St. Louis, MO, USA; Department of Psychiatry, Washington University Medical School, St. Louis, MO, USA; African Mental Health Research and Training Foundation, Nairobi, Kenya; Department of Psychiatry, Washington University Medical School, St. Louis, MO, USA; Department of Psychiatry, Washington University Medical School, St. Louis, MO, USA; Department of Psychiatry, Washington University Medical School, St. Louis, MO, USA; Department of Psychiatry, Washington University Medical School, St. Louis, MO, USA; African Mental Health Research and Training Foundation, Nairobi, Kenya; Department of Psychiatry, University of Nairobi, Nairobi, Kenya

**Keywords:** Kenya, KePROS, clinical high risk, CHR, psychosis, schizophrenia, MRI, EEG, AMP SCZ

## Abstract

**Background and Hypothesis:**

The Accelerating Medicines Partnership Schizophrenia (AMP SCZ) funds a longitudinal study of 43 research sites across 5 continents to develop tools to stratify developmental trajectories of youth at clinical high risk for psychosis (CHR) and identify homogenous targets for future clinical trials. However, there are no sites in Africa, leaving a critical gap in our knowledge of clinical and biological outcomes among CHR individuals.

**Study Design:**

We describe the development of the Kenya Psychosis-Risk Outcomes Study (KePROS), a 5-year NIH-funded project in Kenya designed to harmonize with AMP SCZ. The study will recruit over 100 CHR and 50 healthy participants and conduct multiple clinical and biomarker assessments over 2 years. Capacity building is a key component of the study, including the construction of an electroencephalography (EEG) laboratory and the upgrading of a local 3 T magnetic resonance imaging (MRI) machine. We detail community recruitment, study methodologies and protocols, and unique challenges with this pioneering research in Africa.

**Study Results:**

This paper is descriptive only. Planned future analyses will investigate possible predictors of clinical outcomes and will be compared to results from other global populations.

**Conclusions:**

KePROS will provide the research community with a rich longitudinal clinical and biomarker dataset from an African country in the developing Global South, which can be used alongside AMP SCZ data to delineate CHR outcome groups for future treatment development. Training in mental health assessment and investment in cutting-edge biomarker assessment and other technologies is needed to facilitate the inclusion of African countries in large-scale research consortia.

## Introduction

Schizophrenia has a worldwide prevalence ranging between 0.4% and 1%^1–3^ and is associated with significant burden and societal costs.^4,5^ Lifetime prevalence rates in Africa have been reported as similar or lower than most studies in the “Global North” (eg, Europe, North America, Australia). However, these observations are complicated by there being only a few large-scale epidemiologic studies in Africa.^1,2,6^ Nevertheless, reported schizophrenia rates in Africa are consistent with reduced frequency in developing countries,^7–9^ although this has also been questioned.^10^ Despite this possible reduced prevalence of schizophrenia in Africa, rates are substantially increased among African immigrants to Europe,^11–13^ including in subsequent generations,^13^ and are reported to be 2–3 times higher in Black populations in the United States compared to Caucasians.^14–16^ Furthermore, higher rates of self-reported psychotic experiences have been found among those with African ancestry in the diaspora compared to those with other ancestries.^17,18^ The reasons for these disparities are not entirely clear and have been attributed to biased prevalence estimates as well as unique environmental stressors, including marginalization and real or perceived discrimination.^19–21^ Differential genetic risk has also been considered; however, this seems less likely in the absence of increased schizophrenia prevalence among those living in Africa. Such questions, however, underscore the importance of conducting research in developing countries in general, and in Africa in particular: to provide deeper insights into the causes and risks of schizophrenia and help reduce the burden of the illness in high-risk groups both in Africa and beyond.

Preventing the onset of schizophrenia and related psychotic disorders has been an increasingly studied area of research. In recent decades, efforts to identify the schizophrenia prodrome prospectively have resulted in the development of a research diagnosis commonly referred to as the clinical high risk for psychosis (CHR). Three diagnostic subtypes of the CHR have been proposed, involving either attenuated psychotic experiences, brief episodes of full-blown psychotic symptoms, or genetic risk with recent functional deterioration.^22^ Most individuals meeting CHR criteria experience attenuated psychotic experiences, although those with brief intermittent psychotic episodes have been found to convert to a psychotic disorder at higher rates.^23^ CHR conversion rates have been reported to be between 15% and 30% over 3 years,^24–29^ with greater rates in help-seeking youth,^30^ which is substantially higher than the lifetime prevalence of psychosis in the general population. Among those who do not convert, approximately half will go through remission of symptoms, and the others will either persist or progress in their attenuated psychotic experiences, but without meeting full-blown psychotic symptom criteria.^31^ In addition, as is the case with schizophrenia, there is also substantial heterogeneity in clinical symptoms in those with CHR, which commonly is accompanied by some combination of mood symptoms, anxiety, cognitive impairments, or social difficulties.^32,33^

Over the years, there have been several large-scale CHR studies conducted to better understand the psychopathology and neurobiology associated with CHR, facilitate earlier intervention in at-risk youth, and improve long-term outcomes in schizophrenia and other psychotic disorders.^34,35^ In 2008, the National Institute of Mental Health (NIMH) funded an 8-site consortium, the North American Prodromal Longitudinal Study (NAPLS), with the goal of identifying predictors of psychosis conversion.^36^ Data derived from this study, and its subsequent renewals,^37^ found that an increased risk of conversion to psychosis was associated with genetic risk, higher levels of unusual thought content, social impairment, and disorganized communication.^27,38^ Among biomarker assessments, NAPLS studies have also reported accelerated cortical thinning in prefrontal, temporal, and parietal regions preceding psychosis onset.^39,40^ Additionally, NAPLS has investigated the role of neurophysiological, neurocognitive, and neurohormonal factors in the development of psychosis.^37^ CHR outcomes have also been investigated in other independent studies around the world. Our group conducted the only CHR longitudinal study in Africa to date, an NIH-funded study in Kenya that followed 135 high-risk and 142 low-risk youth over 20 months.^41^ This study found psychosis conversion rates of 3.8%, with only disorganized communication predicting conversion, which was also among the predictors identified by the NAPLS consortium.^38^

Despite progress in the field, a major obstacle in psychosis prevention efforts is the substantial heterogeneity in the clinical course and underlying neurobiological mechanisms of affected individuals and a lack of reliable and valid predictive biomarkers. Models generated from existing studies^36,42–44^ have only shown modest predictive value in estimating clinical trajectories, and therefore have only limited clinical utility on an individual level.^45,46^ Additionally, heterogenous clinical and pathophysiological trajectories of CHR youth hinder intervention studies, which require relatively homogenous subject groups.^47^ More effective methods are therefore needed to stratify individuals based on their clinical and pathophysiological trajectories and to develop reliable prognostic and predictive biomarkers to select primary endpoints and targets for future clinical trials.

Generating tools that will fast-track the development of effective, early-stage treatments for people who are at risk for schizophrenia is the major aim of the Accelerating Medicines Partnership Schizophrenia (AMP SCZ), a public–private partnership between the NIH, the U.S. Food and Drug Administration, the European Medicines Agency, pharmaceutical and life science companies, and non-profit and other organizations.^48,49^ Under the AMP SCZ framework, 2 international networks were funded by the NIH in 2020. The Psychosis-Risk Outcomes Network (ProNET) now comprises 28 sites around the world, including 21 in North America, 5 in Europe, and 2 in Asia. The Prediction Scientific Global Consortium (PRESCIENT) comprises 15 international sites, including 6 in Australia, 3 in Asia, 5 in Europe, and 1 in South America. In addition to these 2 networks, the Psychosis Risk Evaluation, Data Integration, and Computational Technologies—Data Processing, Analysis, and Coordination Center (PREDICT-DPACC) were funded to integrate and process the data generated from the 2 multisite research networks. The AMP SCZ program aims to recruit approximately 2000 CHR participants and 640 matched control participants across 43 sites, and its large, international scope is important for promoting generalizable findings, at least among a broad range of developed countries.

**Fig. 1.**
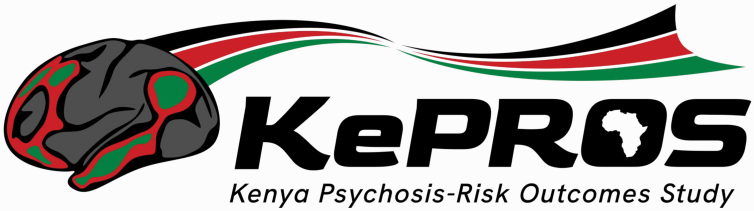
The KePROS logo. The design incorporates a stylized functional MRI-derived default mode network^50^ brain map in the colors of the Kenyan flag. KePROS, Kenya Psychosis-Risk Outcomes Study; MRI, magnetic resonance imaging.

In 2021, a 5-year research award was funded by the NIH, referred to as the Kenya Psychosis-Risk Outcomes Network (KePROS; figure 1). The main justification for KePROS was that among the 45 international AMP SCZ research sites, there were no sites in developing countries, or Africa more specifically, despite the representation of developed countries from every other continent. This was of particular concern considering the aforementioned issues of possibly lower prevalence of schizophrenia in Africa, combined with the observation that rates of schizophrenia in most epidemiologic studies have been highest among those with African ancestry,^11–16^ both of which indicate the possible importance of socioenvironmental and genetic factors in schizophrenia and psychosis. KePROS is led by Washington University in St. Louis (PI: Mamah) in collaboration with the African Mental Health Research and Training Foundation in Nairobi (PI: Ndetei). The project will recruit at least 100 CHR and 50 healthy control individuals from Kenyan communities and model the methods from the multisite AMP SCZ consortia.

Below, we describe the specific organization, assessments, and protocols of KePROS, and highlight challenges, modifications, and capacity-building efforts.

## Methods

### Overview of Study

The KePROS study was developed to closely approximate studies at the 43 AMP SCZ sites to facilitate harmonization and comparison. It is an observational longitudinal study of Kenyan adolescents and young adults, involving both behavioral and biomarker assessments over 2 years. Data collected through KePROS was however not funded to be processed through the PREDICT-DPACC, in contrast to that from the AMP SCZ recruitment sites. All data from KePROS will be made publicly available through an NIH Data Archive (NDA).

KePROS will be conducted by research staff at the Africa Mental Health Research and Training Foundation (AMHRTF), led by its founding director, David Ndetei, MD, PhD, DSc., a leading psychiatrist and researcher in Kenya. The study’s Principal Investigator, Daniel Mamah, MD, is a Professor of Psychiatry at Washington University in St. Louis. The 2 individuals and organizations have a long collaborative research history involving community assessments of CHR youth and those with psychotic-like experiences.^33,41,51–64^ The KePROS principal investigator (D.M.) and magnetic resonance imaging (MRI), electroencephalography (EEG) and digital phenotyping co-investigators (M.H., A.A., and J.T.) have identical roles in the Washington University AMP SCZ research site or in ProNET more broadly, further facilitating the implementation and harmonization of KePROS to AMP SCZ.

The study was approved by The Nairobi Hospital Ethics Research Committee and the Institutional Review Board of Washington University in St. Louis.

### Participants and Recruitment

The study will include a minimum of 100 CHR and 50 matched healthy control participants, aged 13–30 years. Participants will be recruited from 4 communities in or around Nairobi: Kibera, Kiambu, Machakos, and Makueni. The Nairobi neighborhood, Kibera, is the largest urban slum in Africa, with residents living in extreme poverty. Kiambu, Machakos, and Makueni counties include suburban and rural regions and comprise both middle-income and poorer populations.

Recruitment will be modeled after our group’s prior successful community recruitment of CHR participants.^33,41,54,55^ To identify participants potentially eligible for the study, research assistants from AMHRTF plan to screen >5000 youth or young adults, including from secondary schools, universities, and technical colleges. Youth will also be screened from community gathering spaces (eg, large rooms or meeting halls) organized by community leaders. The screening will be done using the Washington Early Recognition Center Affectivity and Psychosis (WERCAP) Screen, a 16-item questionnaire, of which 8 items measure the severity of psychotic experiences based on both symptom frequency and effect on functioning.^55,65,66^ The WERCAP Screen was designed to be cross-culturally applicable and is highly sensitive to even the slightest psychotic-like experiences, showing community prevalence rates of >70%.^53^ A cut-off score of ≥30 will be used to preselect potential CHR participants based on a previous validation study in Kenya predicting CHR status.^55^ Healthy controls (HC) would be preselected based on WERCAP psychosis scores of ≤3, matched by sex, age, geographical location, and tribe.

**Fig. 2.**
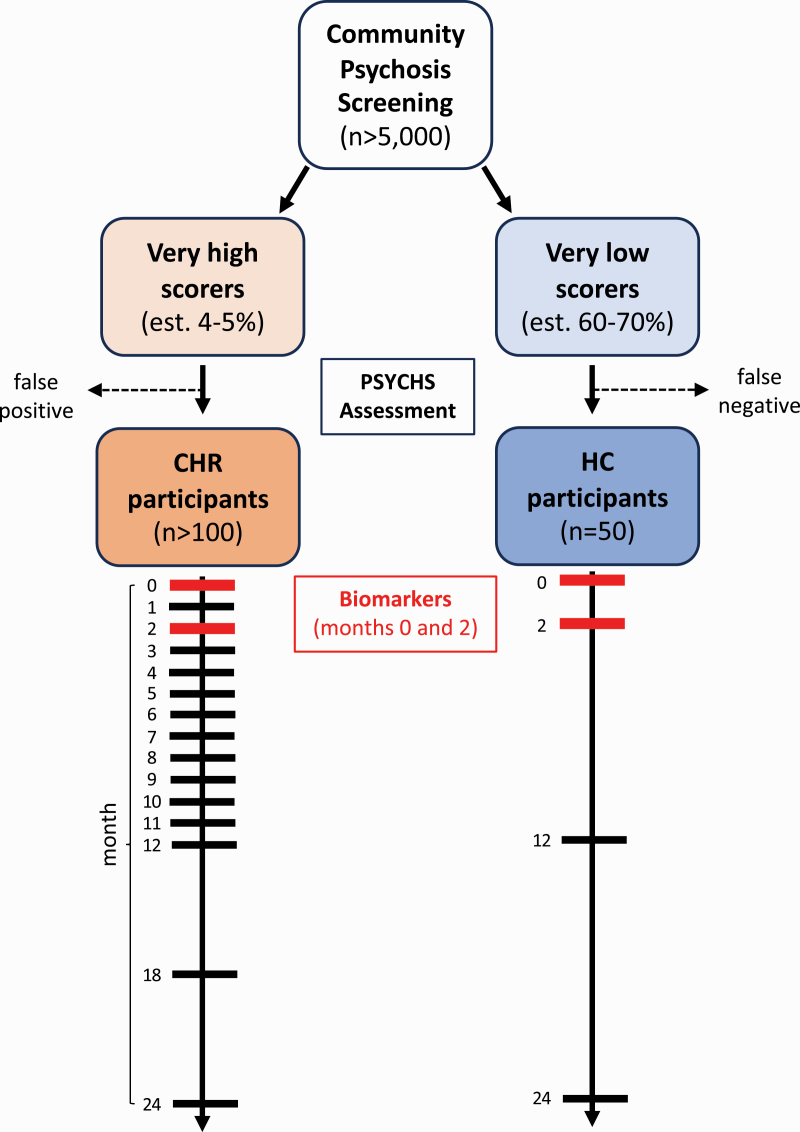
Recruitment and assessment flow chart. Psychosis screening will be done in individuals aged 13–30 years at schools and other designated community settings, using the WERCAP Screen.^55,65^ A score of ≥30 will be used to screen for possible CHR individuals and ≤3 to preselect healthy controls (HC). The PSYCHS^67^ will be used to ascertain the official CHR or HC status in the preselected individuals. Longitudinal assessments will occur over 24 months, with less frequent visits in HC. Biomarker assessments will only be conducted at baseline and month 2. CHR, clinical high risk for psychosis.

The KePROS recruitment and assessment flowchart is shown in figure 2. The final selection of CHR and healthy control participants will be determined using the same structured interview employed by AMP SCZ (PSYCHS^67,68^; discussed below).

The inclusion and exclusion criteria for CHR participants are identical to that of AMP SCZ. They are briefly summarized in table 1 and elaborated more fully in Wannan et al.^49^ Criteria for HC are identical except they also: (a) do not meet CHR criteria or have a Cluster A personality disorder, and (b) do not have a first-degree family history of schizophrenia spectrum disorders. Criteria for psychosis conversion will be determined using the PSYCHS as shown in table 1.

**Table 1.** Inclusion, Exclusion, and Conversion Criteria for CHR Participants

Inclusion criteria
1. Age between 13–30 yrs 2. Meet the criteria for CHR syndrome using the PSYCHS 3. Understand and sign informed consent (assent for minors)
Exclusion criteria
1. Antipsychotic medication exposure equivalent to a total lifetime haloperidol dose of >50 mg 2. Current or past psychotic disorder other than attenuated psychosis syndrome 3. Impaired intellectual functioning (IQ < 70) 4. History of clinically significant neurological disorder 5. Traumatic brain injury (≥7 on Traumatic Brain Injury screening instrument) 6. Ineligibility for MRI scanning (eg, metal objects in body)
Conversion criteria
Meeting the full threshold for a positive psychotic symptom on the PSYCHS, with any of 3 options: 1. Lasting ≥1 week, and occurring either (a) for >1 h/day, 3–7 days/week OR (b) daily for <1 h 2. Lasting <1 week with newly prescribed or newly increased antipsychotic dose, occurring either (a) for >1 h/day, 3–7 days/week OR (b) daily for <1 h 3. Symptom is imminently dangerous

*Note*: CHR, clinical high risk for psychosis; MRI, magnetic resonance imaging; PSYCHS, positive symptoms and diagnostic criteria for the CAARMS harmonized with the SIPS.

### Timeline of Assessments

The schedule of assessments for CHR participants also matches AMP SCZ and is shown in table 2. It includes structured clinical interviews and questionnaires to probe behavior, demographics, and treatment history assessed periodically over 24 months. Biomarker data (including MRI, EEG/ERP [event-related potentials], and fluid data) will be collected at the baseline and month 2 assessments. The major reason for choosing a relatively short interval between biomarker collection time points in AMP SCZ is to provide data for possible early stratification in future clinical trials. Digital markers, including ecological momentary assessments, digital phenotyping, and actigraphy will be collected continually over 12 months for participants who opt-in to those measurements (see below).

**Table 2.**
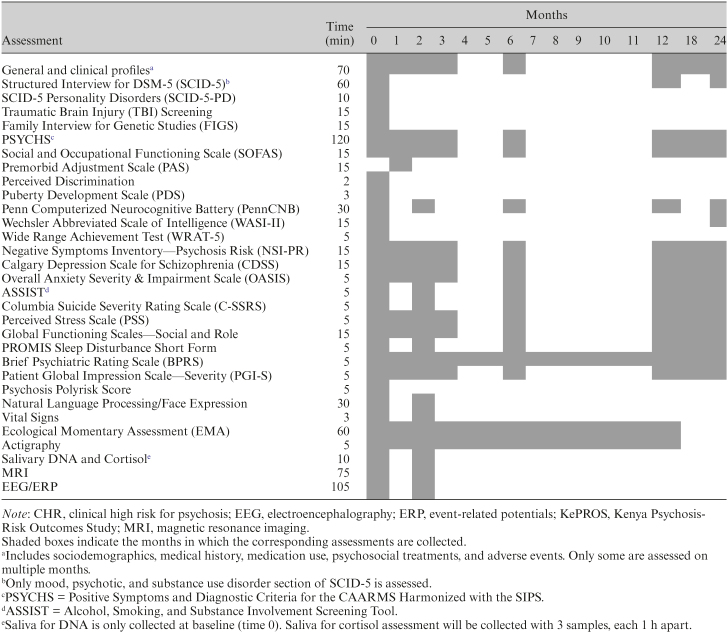
KePROS Timeline of Assessments for CHR Participants

All CHR participants will complete the assessments shown in table 2. Of the healthy control participants, 25 will only complete baseline assessments. The remaining 25 control participants will complete additional key assessments at months 2, 12, and 24. In general, participants will be assessed in person at their recruitment location. Biomarker assessments, natural language processing (NLP), facial expression sampling (FES), and neurocognitive assessments will be collected after transporting participants to Nairobi. When in-person assessments are not feasible, virtual visits (with both video and audio) will be used for assessments that do not require the participants’ physical presence.

### EEG and ERP

**Fig. 3.**
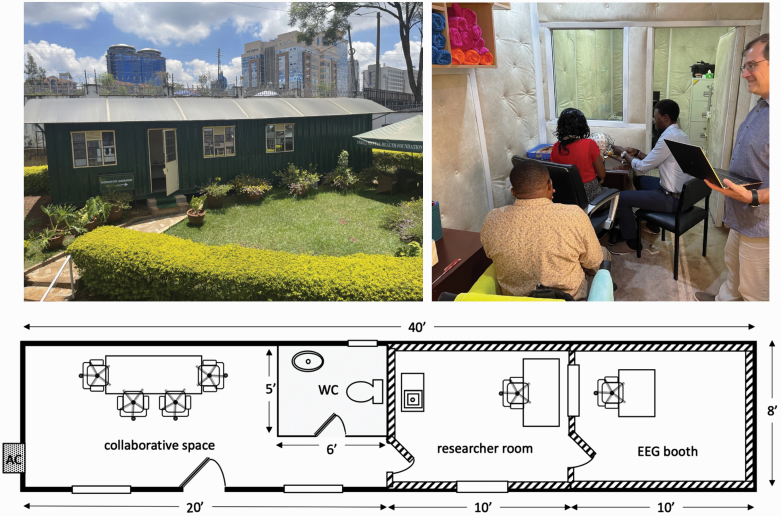
Newly built electrophysiology and biomarker laboratory in Nairobi. The laboratory was constructed using a 40 × 8 ft shipping container on the premises of the African Mental Health Research and Training Foundation. Shown are Kenyan research staff and Dr. Andrey Anokhin, co-investigator (right).

Reliably conducting electrophysiological recordings in KePROS initially presented a challenge, as an adequate-sized secure and soundproof space could not be identified in the area. For this reason, a 40 × 8 ft shipping container was purchased and transported onto the AMHRTF research premises where it was converted into an EEG “biomarker laboratory.” The completed building, which was formally launched on January 9, 2023, includes 2 rooms with heavily padded walls for electrophysiological recordings (figure 3). The remaining research space, which includes a −20°C freezer, would be used for fluid collection and storage, collecting vital signs, and conducting NLP and FES.

KePROS is leasing a 64-channel EEG recording system from Brain Products (actiCHamp), which is identical to that leased by all 43 AMP SCZ sites. It includes preprogrammed assessment modules for (1) resting EEG; (2) gamma oscillations (40 Hz auditory steady-state response)^69^; (3) auditory P300 ERPs to infrequent target tones (P3b) and novel sounds (P3a)^70,71^; (4) an auditory mismatch negativity task to tone duration and pitch during a visual oddball task; and (5) a visual P300 ERP to infrequent target circles (P3b) and novel fractal image (P3a).^70,72^ These assessments are identical to those being collected for AMP SCZ.

On-site and remote EEG/ERP training of Masters and Doctorate-level AMHRTF research staff was done by Dr. Andrey Anokhin, an experienced EEG/ERP co-investigator of the Washington University AMP SCZ site.

### Magnetic Resonance Imaging

Participant brain scans will be collected at The Nairobi Hospital, a 355-bed private hospital in the Upper Hill area of Nairobi, with 2 3 T scanners—a Siemens Verio and Philips Achieva. KePROS is using the Verio, which is a wide-bore (70 cm) system with 45 mT/m peak gradients (200 T/m/s slew rate), first introduced in 2007. Two major software upgrades were applied to the Verio at Nairobi Hospital for the KePROS study. First, a Siemens software upgrade (“Neuro fMRI/DTI Combi Package”) was purchased to enable functionality that Nairobi Hospital had not needed previously as part of its clinical scanning, namely the capability to collect blood oxygen level-dependent (BOLD) acquisitions and diffusion-weighted acquisitions with multi-directional, user-specified directions. Second, multiband (MB) accelerated sequences were obtained for the functional and diffusion MRI (fMRI/dMRI) acquisitions through the Center for Magnetic Resonance (CMRR) at the University of Minnesota. Multiband (ie, simultaneous multi-slice, SMS)^22,23^ permits the acquisition of multiple slices simultaneously, thus facilitating whole brain coverage with smaller voxels and/or shorter repetition times (TRs). Notably, a Siemens product implementation of multiband is not available for the software level (VB17) installed on the Verio at Nairobi Hospital. Further, the CMRR sequences are used for the fMRI and dMRI acquisitions on the Siemens scanners for AMP SCZ, so it was not only necessary but more harmonized with AMP SCZ to use the CMRR MB sequences for KePROS as well.

While a large majority (90%) of AMP SCZ sites are using Siemens scanners, none of them are specifically using the Verio model. (Of note, no Prisma, Skyra, or Vida installations currently exist in Nairobi, which are the Siemens 3 T models involved in AMP SCZ.) But a more important factor, which impacted our ability to simply adopt the AMP SCZ “Tier 2” (Skyra/Vida) protocol, was the fact that the Verio at Nairobi Hospital only has a 16-channel Head/Neck coil (with 12 “head” and 4 “neck” channels). In contrast, the AMP SCZ sites are all using either a 32-channel Head or 64-channel Head/Neck coil. This presented 2 fundamental challenges that we had to consider: (1) inherently lower signal-to-noise (SNR) due to the reduced number of channels in the coil, and (2) the possible need to lower the multiband factor since the number of simultaneous slices that can be successfully unaliased (without artifact) is reduced when fewer coils are spanning the direction of the slices. We considered the possibility of purchasing a 32-ch head coil, but the Verio at Nairobi Hospital has only 18 receiver channels, so an upgrade of the entire radio frequency (RF) chain to 32 channels would have been necessary, which was cost-prohibitive.

**Fig. 4.**
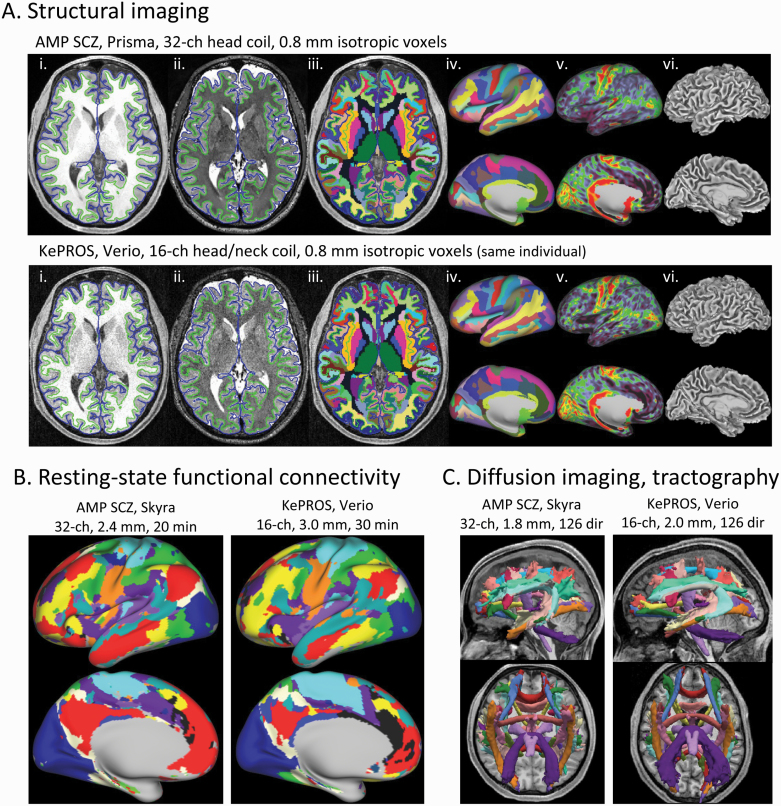
Comparing brain images and derived results for AMP SCZ vs KePROS protocol. (A) Panels (i)–(vi) illustrate the strong similarity of structural-derived results from a 51-year-old healthy male scanned on the Prisma at Washington University using the AMP SCZ protocol (top row), compared to that same individual scanned on the Verio at The Nairobi Hospital using the KePROS structural protocol (bottom row). (i) Axial slice of T1w scan, (ii) T2w scan, and (iii) FreeSurfer’s “wmparc” volumetric segmentation, each with the FreeSurfer “pial” (blue) and “white” (green) surface contours overlaid; (iv) Freesurfer’s “aparc.a2009s” surface segmentation displayed on “very inflated” left hemisphere lateral and medial surfaces; (v) smoothed T1w/T2w ratio (“myelin”) map (“SmoothedMyelinMap_BC”); (vi) mid-thickness surface. Results were generated using the HCPpipelines.^91^ While there is a noticeable difference in image SNR for the same 0.8 mm isotropic spatial resolution (due to the 16-ch, rather than 32-ch, coil), the quality of the KePROS structural acquisition is sufficient for accurate surface generation and yields highly similar segmentations and myelin maps. (B) Results from 2 different individuals of resting-state functional connectivity network maps generated using network template matching,^92^ with a small amount (6 mm full-width-half-maximum) of surface-based spatial smoothing applied to the input data. The AMP SCZ fMRI scans were acquired from a 20-year-old female healthy control participant on a Skyra scanner using the AMP SCZ fMRI protocol (ie, 20 total minutes with 2.4 mm voxels and MB factor of 6). The KePROS fMRI scans were acquired from a 24-year-old male CHR participant using the KePROS fMRI protocol (ie, 30 total minutes with 3 mm voxels and MB factor of 3). Note that the KePROS and AMP SCZ examples shown have comparable overall quality of their individually derived connectivity network maps. (C) Results from the same 2 individuals shown in (B) of TRACULA analyses of white matter tracts^93^ generated from the diffusion-weighted scans of the respective protocols (top: left view; bottom: inferior view). AMP SCZ “Tier 2” (Skyra) diffusion protocol was acquired over 10 min with 1.8 mm isotropic voxels, MB factor of 3, and 126 diffusion directions (4 shells with *b* = 200, 500, 1000, and 2000 s/mm^2^). KePROS was acquired over 12 min with 2 mm isotropic voxels, MB factor of 2, and the same diffusion directions and *b*-values. The TRACULA results are of comparable quality. See table 4 for further protocol details. AMP SCZ, Accelerated Medicine Partnership Schizophrenia; CHR, clinical high risk for psychosis; KePROS, Kenya Psychosis-Risk Outcomes Study.

The KePROS imaging protocol collects all the same MR modalities as AMP SCZ, namely T1-weighted and T2-weighted structural scans, multiple runs of “resting-state” BOLD fMRI, and the same “Tier 2” diffusion imaging protocol collected at the non-Prisma sites in AMP SCZ (table 3). But as a consequence of the above hardware limitations, several modifications of the AMP SCZ imaging protocol were necessary for KePROS, to maintain appropriate SNR and image quality, without significantly increasing participant burden (ie, substantially longer scan durations). These modifications are shown and explained further in table 4. Some comparisons of scans and results from KePROS relative to AMP SCZ are shown in figure 4.

**Table 3.** KePROS MRI Scanning Protocol

MRI Scan	Duration (min:s)^a^
Localizer/AutoAlign block	0:44
Field Map pair^b^	0:36
Resting-state fMRI run 1 (“AP” polarity)	7:36
Resting-state fMRI run 2 (“PA”)	7:36
Field Map pair	0:36
T1w structural (MPRAGE)	6:54
T2w structural (SPACE)	5:34
dMRI block^c^	12:12
Field Map pair	0:36
Resting-state fMRI run 3 (“AP”)	7:36
Resting-state fMRI run 4 (“PA”)	7:36
Total	57:36

*Note*: KePROS, Kenya Psychosis-Risk Outcomes Study; MRI, magnetic resonance imaging.

All scans are collected using Siemen’s AutoAlign functionality, for automated positioning of the field-of-view position and orientation, which simplifies the scanning session and ensures consistent positioning across individuals.

^a^As reported by the scanner; includes any initial calibration scans or discarded frames.

^b^A pair of spin-echo EPI scans with opposite phase encoding polarity (“AP” and “PA”), exactly matched to the fMRI scans on field-of-view, voxel and matrix size, bandwidth, and echo spacing. Used to estimate the local B0 field inhomogeneity and correct for susceptibility distortion in the fMRI scans.

^c^Consists of a 126-direction “main” dMRI scan (10:32) acquired with PA polarity, with preceding and following scans with 6 “b=0” volumes with AP polarity (each 0:50), for estimation and correction of susceptibility distortion.

**Table 4.** Comparison of KepROS and AMP SCZ Imaging Protocols, Including Key Differences

MR Parameter	KePROS	AMP SCZ (Skyra, “Tier 2” version)^a^
RF coil	**16-ch Head/Neck** (with neck elements on)^b^	**32-ch Head**
T1w MPRAGE		
Voxel size (mm isotropic)	0.8^c^	0.8
TR/ TE/ TI (ms)	2500/2.07/1000	2500/2.07/1000
Flip angle (deg)	8	8
Bandwidth (Hz/Px)/echo spacing (ms)	240/7.6	240/7.6
T2w SPACE		
Voxel size (mm isotropic)	0.8^c^	0.8
TR/ TE (ms)	3200/567	3200/564
Bandwidth (Hz/Px)/Echo Spacing (ms)	744/3.6	679/3.86
Duration (min:s)	5:34^d^	5:57
fMRI (using CMRR MB “bold” sequence)		
Voxel size (mm isotropic)	**3.0** ^e^	**2.4**
MB factor	**3** ^e^	**6**
TR/TE (ms)	1110/30	900/35
Flip angle (deg)	60	52
Bandwidth (Hz/Px)/echo spacing (ms)	2394/0.52	2136/0.61
Measurements (frames)/duration (both per run; 4 runs total)	**405/7:30**	**333/5:00**
dMRI (using CMRR MB “diff” sequence)		
Voxel size (mm isotropic)	**2.0** ^e^	**1.8**
MB factor	**2** ^e^	**3**
TR/TE (ms)	**4850/91.4**	**3970/96.6**
Bandwidth (Hz/Px)/echo spacing (ms)	1714/0.69	1602/0.73
# Diffusion directions	126^f^	126
Duration (min:s) of dMRI block	12:12	10:12

*Note*: AMP SCZ, Accelerated Medicine Partnership Schizophrenia; KePROS, Kenya Psychosis-Risk Outcomes Study; MR, multiband; RF, radio frequency; TE, echo time; TI, inversion time; TR, repetition time.

Values in bold reflect a qualitative indication of key differences between the KePROS and AMP SCZ versions.

^a^We compare specifically to the Skyra protocol in AMP SCZ, since the Skyra has 45 mT/m gradients, like the Verio.

^b^The neck elements were activated for all scans, since piloting indicated a small improvement in image quality in the inferior brain with them enabled.

^c^0.8 mm structural scans with a 16-ch Head/Neck coil clearly have reduced SNR relative to what is routinely obtained using a 32-ch head coil. However, we considered the SNR sufficient for accurate segmentation and surface generation. This will be monitored in the initial KePROS participants and an adjustment to spatial resolution implemented if necessary.

^d^We suspect the 23 s difference in scan duration in the T2w scan (despite identical TR and matrix size) reflects a subtle difference in the precise implementation of the k-space trajectory of the variable flip angle SPACE sequence between the VB17 (KePROS) and VE11 (AMP SCZ) software lines.

^e^Voxel size was increased and MB factor decreased in KePROS relative to AMP SCZ to accommodate the limitations of a 16-ch coil. The larger voxels help restore SNR that is lost due to the lower overall SNR of a 16-ch coil, and the reduced MB factor avoids poorer quality reconstructions (ie, unaliasing artifacts) that result if too high of an MB factor is used in the presence of a reduced number of coil elements in the direction of the slice plane.

^f^Identical diffusion directions between KePROS and the AMP SCZ “Tier 2” (ie, non-Prisma) dMRI protocol. It is a multi-shell acquisition, with interleaved b-values of 200, 500, 1000, and 2000 s/mm^2^, with *n* = 6, 10, 50, and 50 directions in each shell, respectively, plus 10 interleaved *b* = 0 acquisitions.

On-site development, testing, and instruction of the MRI protocol was done by Dr. Michael Harms, a co-investigator of the Washington University AMP SCZ site and co-leader of the neuroimaging team for AMP SCZ.

### Digital Health Technologies-Derived Measures

As with AMP SCZ, participating in digital assessments in KePROS is an optional aspect of KePROS. For those who participate in these assessments, the duration of participation is up to 1 year, with the frequency of assessment indicated below. The specific digital methodologies used in the study include:

(1) *Ecological momentary assessment (mobile phone surveys)*: Phone-based self-report questionnaires will be collected using the open-source mindLAMP app, compatible with both Android and Apple smartphones, developed by John Torous.^73^ Notably, mindLAMP works offline, so internet connectivity is only needed for initial setup and to transmit data off the app to study servers. All questions will be the same as those used in AMP SCZ. Participants will be sent multiple brief questions daily about their thoughts, feelings, and behaviors across multiple domains. Participants who do not have mobile phones with the capability to support this software will be provided one. Cellular cards will also be provided when needed in low-coverage areas since Wi-Fi or a data plan is required for data transfer of the mobile phone. Mobile phones are prohibited in most Kenyan high schools; thus, this data may not be obtained consistently from this population. Use of the mindLAMP app will be frequently monitored and reviewed for quality by its development team at Beth Israel Deaconess Medical Center at Harvard, Dr. John Torous, and research staff.
(2) *Digital phenotyping (geolocation, accelerometer, and screen state)*: The mindLAMP app also has the option to collect passive sensing data, namely geolocation, accelerometer, and/or screen state (on/off) for the participants who opt-in to share this data. This data can be used to infer patterns of sleep duration, sedentary periods, mobility patterns, timing and duration of smartphone screen exposure, exposure to green space, time in urban environments, and other novel markers created from combining features.
(3) *Actigraphy (activity monitoring)*: Participants’ daily activity will be assessed 24 h for up to a day for a year using a wearable wrist device, Axivity AX3 (Newcastle upon Tyne, UK).

### DNA and Cortisol

In KePROS, blood will not be collected to minimize culture-driven apprehension of study participation and risks associated with blood draws. As the primary reason for blood collection in AMP SCZ is to obtain DNA, genetic material will instead be obtained from saliva, which imparts minimal risk to data quality.^74^ Salivary DNA will be collected using Oragene saliva kits (DNA Genotek, Stittsville, ON), as previously done by our group in Kenya. Saliva kits will be sent to Washington University’s Hope Center DNA/RNA purification facility for storage.

Saliva for cortisol assessment will be collected in kits (Salimetrics, USA) with 3 samples, each an hour apart using passive drool and stored at −20°C. Kits will be shipped to Salimetrics for analysis.

### NLP and FES

(1) *Natural Language Processing:* Spoken English language will be captured over the first 20 min of the semi-structured PSYCHS interview administration using Zoom’s online meeting platform. Spoken language samples will also be obtained from smartphone audio diaries. Specialized software will be used to extract various acoustic features such as voice stability, pitch variations, timbre, and unique characteristics.^75^ Language samples will be converted into text using an automated transcription service (TranscribeMe!^76^), and linguistic features will be analyzed.
(2) *Facial Expression Sampling:* This will also be captured during the semi-structured PSYCHS interviews. Specialized software will extract facial action units, to enable the detection of common facial expressions and emotions.^77^

Both NLP and FES will be conducted in the enclosed biomarker laboratory at the premises of AMHRTF in Nairobi, to maximize privacy. All audio and video data will be encrypted and stored securely, removing all personal identifiers, on an online platform, as in AMP SCZ study sites.

## Discussion

The Kenya Psychosis-Risk Outcomes Study (KePROS) is the most comprehensive longitudinal study of CHR populations ever conducted in Africa. It comprises elaborate assessments, and for the first time involves specific biomarkers, including MRI and electrophysiological investigations.^48^ While KePROS is not formally part of the large multisite AMP SCZ international cohort, it includes many identical assessments (the primary difference being some modifications necessary to the MRI protocol to accommodate hardware limitations) and comprises multiple individuals who overlap with the AMP SCZ Washington University research site, including the principal investigator, MRI and EEG co-investigators, research staff, and the digital phenotyping core director of AMP SCZ. This structure ensures that the research community (after public sharing) receives a broad array of data that can be validly compared with that from other global populations.

Another benefit of KePROS is its research capacity building in Kenya, particularly as regards biomarker research capacity. Training of local research staff in administering EEG and ERP assessments, and the newly built EEG and biomarker laboratory in Nairobi will incentivize future investigations in this area, beyond the current study. Similarly, upgrading a local MRI scanner with applications that permit sophisticated multimodal neuroimaging and training radiographers will facilitate advanced research involving brain structure and function. This expertise will be valuable for subsequent research studies in other psychiatric and neurological populations. Such studies are novel in most parts of Africa, and KePROS’ efforts have been enthusiastically received by hospital administrators and government officials. KePROS has also generated substantial interest from students, physicians, and researchers, which bodes well for generating future research into psychosis by new investigators.

KePROS represents the second time CHR youth will be longitudinally evaluated in Kenya, following an earlier study by our group in which clinical measures alone were assessed.^41^ While no biomarkers were collected in that study, 135 psychosis-risk individuals were followed for 20 months and revealed a 3.8% conversion rate, with only baseline disorganized communication predicting conversion.^41^ This conversion rate was lower than most other global CHR studies and may be attributable to a lower average participant age, shorter study duration, and recruitment of a non-help-seeking population. A pilot study of 6 CHR patients in Tunisia resulted in 1 conversion after 3 months, which would be a 17% conversion rate if maintained over a larger cohort.^78^ Other CHR studies in Africa have included investigations of neurocognitive traits in Kenya, which showed significant impairment primarily in verbal intelligence^54^; investigations of personality traits in Kenya, showing similar attributes as that seen with schizophrenia, with high novelty seeking, low self-directedness and high self-transcendence^33^; and validation studies of psychosis-risk questionnaires in Kenya,^55^ Nigeria,^79^ and Tunisia.^80^

Despite the significant advancement of CHR research through KePROS, there are limitations to the study. The hardware of the 3 T magnet used for scanning is less performant than those used in other AMP SCZ sites, which would result in lower signal-to-noise ratios and poorer image quality if the AMP SCZ protocol was implemented exactly. This is a general challenge in Africa, where there are relatively few MRI scanners, and those that exist typically involve older models and software, making it challenging to implement the latest cutting-edge research protocols deployed in the developed Global North.^81,82^ Substantial investment in MRI technology is therefore necessary in the continent, particularly for newer 3 T magnets with 32 or more RF channels and high channel head coils. Ideally, technological investment should come from academic institutions, but the enormous cost, including maintenance, creates a major challenge. It is unlikely to come from private or public hospitals, as high-end research-oriented scanners are not cost-effective for clinical use. However, with increasing interest in conducting neuroimaging research in Africa, particularly by foreign collaborators, private and public organizations may be incentivized to invest in advanced MRI scanners. Secondly, data from KePROS will not flow through the same DPACC processing pipelines as those from other AMP SCZ sites, which may cause minimal differences in the final processed data that are publicly shared, although we will work to minimize these differences. This is more likely to affect biomarker data than clinical data. Thirdly, data collected through KePROS cannot be generalized to all of Africa, as substantial cultural and biological differences exist across the continent. Thus, expanding CHR studies across Africa is necessary for a more accurate representation of the characteristics of psychosis risk across the continent, which would also provide additional research benefits. For example, research studies in South Africa, which to our knowledge currently possesses the most advanced MR installation in the continent, a 32-channel Siemens 3 T Skyra at the University of Capetown, would allow direct comparison to some of the AMP sites (3 of which are using the Skyra). Also, future CHR studies in the West African country of Ghana, where psychosis risk research is increasing,^83,84^ may provide the closest general approximation with the genetic ancestry of African Americans.

With the considerable growth in CHR research around the world, the relative dearth of similar studies in Africa is striking,^85,86^ highlighting the need to understand the trajectory of high-risk states across different populations, cultures, and socioeconomic environments. It remains challenging to conduct CHR studies in developing countries, which has been attributed to insufficient funding, difficulties gathering longitudinal data, and cultural barriers and stigma.^87^ Early identification of psychosis risk promises improved outcomes,^34,35^ which is particularly important in many parts of Africa where appropriate treatment of schizophrenia is infrequent due to health systems with underfunded or nonexistent mental health programs.^88^ Earlier interventions would include closer monitoring, psychoeducation, stress reduction, cognitive behavioral psychotherapy and support, and when needed, pharmacological interventions.^89,90^ Such methods, if appropriately carried out, would be cost-effective, by reducing hospital stays, medication costs, and lost productivity. Altogether, in both Kenya specifically and Africa more broadly, there is simultaneously a great need to improve mental health infrastructure and great promise for conducting psychosis and CHR research.
